# The heat is on: sensitivity of goldsinny wrasse to global climate change

**DOI:** 10.1093/conphys/coae068

**Published:** 2024-10-08

**Authors:** Diana Perry, Elena Tamarit, Daniel Morgenroth, Albin Gräns, Joachim Sturve, Martin Gullström, Peter Thor, Håkan Wennhage

**Affiliations:** Department of Aquatic Resources, Swedish University of Agricultural Sciences, Turistgatan 5, 453 30 Lysekil, Sweden; Department of Earth Sciences, University of Gothenburg, Hörsalsvägen 9, 412 58 Gothenburg, Sweden; Department of Applied Animal Science and Welfare, Swedish University of Agricultural Sciences, Medicinaregatan 7B, 41390 Gothenburg, Sweden; Department of Applied Animal Science and Welfare, Swedish University of Agricultural Sciences, Medicinaregatan 7B, 41390 Gothenburg, Sweden; Department of Biological and Environmental Sciences, University of Gothenburg, Medicinaregatan 7B, 413 90 Gothenburg, Sweden; School of Natural Sciences, Technology and Environmental Studies, Södertörn University, S-141 89 Huddinge, Sweden; Department of Aquatic Resources, Swedish University of Agricultural Sciences, Turistgatan 5, 453 30 Lysekil, Sweden; Department of Aquatic Resources, Swedish University of Agricultural Sciences, Turistgatan 5, 453 30 Lysekil, Sweden

**Keywords:** Climate change, *Ctenolabrus rupestris*, cumulative effects, marine heatwave, multi-stressor experiment, ocean acidification, physiology, salinity

## Abstract

Unsustainable harvesting practices have drastically reduced fish populations globally and developments in aquaculture have increased. Unexpectedly, Atlantic salmon farming caused the opening of a new fishery in northern European countries, where previously unharvested mesopredatory species, like the goldsinny wrasse (*Ctenolabrus rupestris*), are captured for use as cleaner fish in pens along the coast and fjords. The goldsinny wrasse is widespread in coastal areas where it plays an ecologically important role as a predator of small invertebrates. Since climate change effects are particularly pronounced in coastal waters, it becomes urgent to understand how fish like the goldsinny will respond to global climate change, including the increasing frequency and intensity of marine heatwaves (MHWs), ocean freshening (OF) and ocean acidification (OA). To address this, we conducted a multi-stressor experiment exposing adult goldsinny to each stressor individually, as well as to all three combined. The results indicated that the goldsinny is highly affected by MHWs and extremely sensitive to a multi-stressor environment, with 34% and 53% mortality, respectively. Additionally, exposure to a MHW event, OF and multi-stressor conditions affected fish metabolism, with the highest standard metabolic- and maximum metabolic-oxygen consumption rates observed for the MHW treatment. Increases in oxidized glutathione (GSSG) and percent oxidized glutathione (% GSSG) in the livers, indicative of oxidative stress, were also seen in the MHW, OF and multi-stressor treatments. As a single stressor, OA showed no significant impacts on the measured parameters. This information is important for conservation of coastal marine environments, given the species’ important role in shallow-water habitats and for management of goldsinny or other mesopredatory fish harvested in coastal ecosystems. The sensitivity of the goldsinny wrasse to future stressors is of concern, and any potential reductions in abundance as a result of climate change may lead to cascade effects with ecosystem-wide consequences.

## Introduction

The goldsinny wrasse (*Ctenolabrus rupestris*) is an important, highly abundant mesopredator with a variable diet consisting of many taxa but dominated by amphipods, copepods, gastropods and bivalves (Wennhage & Pihl, 2002). The species inhabits rocky shores from 1 to 50 m depth, often associated with macroalgae and seagrass beds (Costello, 1991; Pihl & Wennhage, 2002; Stål *et al*., 2007; Perry *et al*., 2018b; Halvorsen *et al*., 2021). The species is widely distributed from Morocco to Norway, naturally occurring within a broad temperature span (Froese & Pauly, 2023) with colder coastal temperatures in Norway of approximately 4°C to warmer summer temperatures of around 22°C in Morocco. Given the variability of coastal environments with large shifts in water temperature and salinity (e.g. Pihl & Rosenberg, 1982; Albretsen *et al*., 2012; Ceccaldi *et al*., 2020), species inhabiting these areas, such as *C. rupestris*, are typically thought to be quite resilient to environmental changes. However, coastal species are currently experiencing an unprecedented degree of change resulting from a myriad of anthropogenic disturbances, which have the potential to reduce ecosystem and species resilience to stressors. The increased Norwegian fishery for *C. rupestris*, to be used as cleaner fish in salmon and rainbow trout farming, has resulted in reduced abundance and body size (Halvorsen *et al*., 2017), which may affect the species’ resilience to climate change.

Norway is the world’s largest producer of Atlantic salmon, and an escalating demand for cleaner fish in the Norwegian salmon farms (>50 million every year) has led to the establishment of a wrasse fishery in Sweden, beginning in 2010, with nearly a million individuals caught and sold from the Swedish west coast in 2013 (Andersson *et al*., 2021). Similar to the Norwegian fishery, the Swedish wrasse fishery focuses on the catch of ballan, corkwing and goldsinny wrasses. Among these species, the highest catch numbers are typically observed for the corkwing and goldsinny wrasses. Unfortunately, evidence shows that cleaner fish in general experience poor welfare and high mortality in the sea cages (Overton *et al*., 2020). In addition, fish caught along the Swedish west coast and transported to the Norwegian salmon farms also constitute a risk of introduction or spread of disease, and escapees from the net pens are a source of genetic variability that could be detrimental to local populations (Faust *et al*., 2018; Bourlat *et al*., 2021). Given that goldsinny wrasse defend nests and show very high site fidelity (Costello, 1991; Cresci *et al*., 2021), the species is exposed to any environmental changes occurring in shallow coastal environments, and are therefore thought to be tolerant to environmental changes. However, we are now entering a time of unprecedented ocean change (Halpern *et al*., 2019).

While nearshore shallow waters experience the largest natural variations in temperature, salinity and pH (e.g. Hofmann *et al*., 2011), they are now also subject to increasingly faster changes. Along the Swedish Skagerrak coast, winter surface water (c. 0–10 m) temperature is increasing by 0.36°C per decade (Thor *et al*., 2023), which is four times as fast as the ocean average. This overall increase in sea surface temperature is accompanied by an increase in the occurrence of marine heatwaves (MHWs) over the last decades (Oliver *et al*., 2018; Spillman *et al*., 2021; Wernberg *et al*., 2021; Stuart *et al*., 2022). Moreover, increasing freshwater runoff is changing the chemistry of coastal waters. On the Skagerrak coast, the average salinity is decreasing by 0.32 units per decade (data from Swedish Meteorological and Hydrological Institute) causing ocean freshening (OF) and this trend is predicted to continue due to increased precipitation, as well as increased recirculation from the fresher waters of the Baltic Sea (Gröger *et al*., 2019; Wåhlström *et al*., 2022). Globally, there is evidence of shifts in ocean chemistry creating more acidified seas (Global Ocean Acidification Observing Network, GOA-ON, 2023; Ocean Acidification International Coordination Centre, OA-ICC, 2023), with ocean acidification (OA) particularly pronounced in nearshore areas (Hofmann *et al*., 2011). Along with these climate-driven changes, many coastal waters experience habitat loss, habitat destruction, and overfishing leading to alterations in community compositions and functioning in coastal habitats (Baden *et al*., 2003, 2012; Cardinale & Svedäng, 2004; Moksnes *et al*., 2008). Along the Swedish Skagerrak coast, the changes already experienced have impacted the coastal marine ecosystems with a shift towards a more mesopredator-dominated system (Perry *et al*., 2018b), leading to an increased importance of species such as wrasses (Bergström *et al*., 2016). These changes have, in turn, potentially reduced the resilience of the system due to species assemblage shifts, making these habitats more vulnerable to further changes. In light of the expected future changes in the region, and the already impacted ecosystem, it is critical to understand how the physiology of ecologically important species, such as the goldsinny wrasse, will respond to forthcoming marine conditions. In addition, given the new targeted fishery for goldsinny wrasse, it is important both ecologically and economically to understand the species’ response to projected climate change.

Climate change influences many physiological functions of marine fish. Fish exposed to heat generally display an elevated metabolic rate (often approximated as oxygen consumption rate using respirometry) as a direct consequence of the effect of temperature on the rate of enzymatic reactions (Lankford *et al*., 2005). Metabolism operates within certain boundaries so that environmental changes, such as warming, first increase metabolic performance (e.g. aerobic scope, the difference between maximum and maintenance level aerobic metabolism) until an optimum beyond which metabolic performance deteriorates (Gräns *et al*., 2014; Pörtner *et al*., 2017). If metabolism increases, reactive oxygen species (ROS) production increases too due to leakage in the electron transport chain. This is generally followed by a response from the antioxidant system of the fish, yet, if the generation of ROS exceeds the capacity of defence, oxidative stress may lead to damage to proteins, lipids and DNA (Finkel & Holbrook, 2000). Thus, long-term physiological performance, as well as survival, is partly reliant upon antioxidant responses as indicated by changes in the activity of antioxidant enzymes (Perry *et al*., 2019, 2024), with exacerbated ROS production leading to physiological damage (Herbert & Steffensen, 2005; Carney Almroth *et al*., 2008; Hernroth *et al*., 2012). Additionally, oxidative stress can result from changes in the aquatic environment other than shifts in water temperature such as salinity and pH (Hellou *et al*., 2012).

In this study, we aimed to (i) evaluate if oxygen consumption rates and antioxidant enzymes and molecules indicative of ROS production are affected by global climate change drivers in goldsinny wrasse, and if so, (ii) determine if cumulative effects of combined stressors can be seen in a multi-stressor environment. Climate change can induce positive, negative or neutral changes in organisms and effects can vary when all environmental drivers are in concert; however, when the individual’s response is negative a driver can become a stress for the organism. We therefore evaluated physiological effects of reduced salinity (OF), increased water temperature (MHW), reduced pH (OA), and all these stressors combined in goldsinny wrasse from the Swedish west coast. Specifically, we studied fish biometric parameters, oxygen consumption and oxidative stress parameters. OF, MHWs and OA were selected because they have been identified as potentially deleterious and are likely to become stressors for fish species (Nissling *et al*., 2002; Ustups *et al*., 2013; Nagelkerken *et al*., 2015; Nagelkerken & Connell, 2015; Perry *et al*., 2019), and are also environmental drivers expected to change in the future within Swedish waters (Meier *et al*., 2012, 2022; Vuorinen *et al*., 2015; Havenhand & Dahlgren, 2017; Gröger *et al*., 2019; Wåhlström *et al*., 2022). The intention is that these results will aid in supplying necessary information to marine managers to create holistic ecosystem-based plans in the face of global climate change.

## Materials and Methods

### Animals and study area

A total of 160 adult goldsinny wrasse (*Ctenolabrus rupestris*) individuals (10.6 ± 1.0 cm SD, 15.9 ± 5.2 g SD) were collected using wrasse cages from the Gullmar Fjord on the Swedish west coast in May 2022 and transported to the laboratory facilities at the Kristineberg Center in Fiskebäckskil (58°14′55.9” N, 11°26′37.4″ E), where they were used in the experiment. No differences were found in fish weight or length among treatment groups at the start of the exposure period (weight H [4, 159] = 5.83, p = 0.21, length H [4, 159] = 3.66, p = 0.45). All animal husbandry and experimental conditions were approved by the Swedish Board of Agriculture’s ethical committee in Gothenburg (permit Dnr 5.8.18–17 034/2021). The fish were kept in tanks with a 13 h:11 h light:dark cycle, and with ambient flow-through seawater pumped in from a depth of 7 m from the sea outside the laboratory (approximately 11°C, 23 salinity, and 8 pH). They were fed daily with thawed shrimp, and excess food was cleaned from the tanks approximately once per week. Fish were held in acclimation in ambient conditions for 5 days prior to the start of the experimental period.

### Experimental design and exposure

All fish were weighed, measured, pit-tagged, read using the Global Pocket Reader Plus (Biomark, Idaho, USA) and randomly placed in cylindrical 200-litre tanks. There were five treatments, each with four replicate tanks, for a total of 20 tanks. Each tank contained eight fish, for a total of 32 fish per treatment and 160 fish in total. The experimental exposure to different treatments lasted for approximately 4 weeks in May–June of 2022 in two thermo-constant rooms at the research facility.

The experimental treatments were control, low salinity, high temperature, low pH and the three stressors combined (multiple stressors). While this terminology is used in the method and result sections of this paper, the treatments simulate the global environmental change drivers of OF, MHW, OA and a multi-stressor ocean. The control treatment tanks were kept at ambient conditions, with a temperature of 13°C, a salinity of approximately 28, and a pH of 8.2 (Table 1). Target experimental values for each treatment were set by taking the last five years of data (2017–2021) from actual water conditions around the study area at the time of the experiment (https://www.weather.mi.gu.se/kristineberg/en/) and then adding/subtracting the appropriate regional end-of-century expected change values (see Perry *et al.*, 2024 for further details). These values were based on the Swedish Meteorological and Hydrological Institute’s (SMHIs) latest regional model predictions based on the Rossby Centre Atmosphere Ocean model (RCAO) coupled with the Swedish Coastal and Ocean Biogeochemical model (SCOBI) (Wåhlström *et al.*, 2022). This set our experimental treatment target values at a salinity of 17 (low salinity), water temperature of 19°C (high temp) and a pH of 7.5 (low pH). For each treatment (except for the multi-stressor treatment), the two other parameters were kept at ambient conditions. For the multi-stressor treatment, the tanks were exposed to all three global climate change drivers (OF, MHW and OA) simultaneously for the full duration of the experimental period. We recorded daily observations of fish welfare and seawater conditions. Temperature was controlled with computerized heat exchangers in the two separate thermo-constant rooms and increased by approximately 1°C per day until the treatment target value was reached. Salinity was decreased by centrally mixed freshwater into the seawater input. The pH was maintained with pure carbon dioxide bubbled into the tanks using a feedback pH-stat computer system (Aqua Medic, Bissendorf, Germany). Both salinity and pH values were set at target treatment values directly at the start of exposure.

**Table 1:** Carbonate chemistry water measurements taken twice weekly throughout the 4-week experiment. The pH is calculated on the pH total scale

	Ambient pH		Low pH		Control Temp		High Temp		Ambient Sal.		Low Sal.			
Parameter	n	mean ± SD	n	mean ± SD	n	mean ± SD	n	mean ± SD	n	mean ± SD	n	mean ± SD	F-ratio var.	P
Alkalinity (μmol kg −1)	60	2093 ± 254	40	2057 ± 252	-	-	-	-	-	-	-	-	1.02	0.48
pH	36	8.23 ± 0.09	24	7.57 ± 0.23	-	-	-	-	-	-	-	-	6.78	<0.001
*p*CO_2_ (μatm)	36	257 ± 69	24	1527 ± 791	-	-	-	-	-	-	-	-	130	<0.001
Temp (°C)	-	-	-	-	60	13.29 ± 0.44	40	18.47 ± 1.35	-	-	-	-	9.33	<0.01
Salinity	-	-	-	-	-	-	-	-	60	27.81 ± 2.45	40	17.18 ± 0.55	19.95	<0.01

Note: Dashes indicate values not analysed

To monitor the conditions throughout the experiment, water temperature, salinity, pH and oxygen were measured 1–3 times per week using a WTW Multi 3430 pH meter. Additionally, water chemistry conditions were recorded approximately twice a week, and subsequently water samples were collected for alkalinity measurements using the TA05 plus/TW alpha plus, SI Analytics (Mainz, Germany) machine (Table 1)*.*

The ambient pH groups differed significantly from the low pH treatments (t(58) = −15, p < 0.001). The ambient pH groups had a pH_TS_ of 8.23 ± 0.09 (*p*CO2 of 256 ± 69 μatm), while the low pH treatments had a pH_TS_ of 7.57 ± 0.23 (*p*CO2 of 1527 ± 791 μatm). Note that while water samples were collected and alkalinity measurements run, pH on the total pH scale could only be calculated for 3 of the 5 sampling days (n = 36 for ambient pH and n = 24 for low pH). See Perry *et al.* (2019) for a detailed description of the alkalinity sampling method*.*

### Respirometry

Fish were individually placed into one of eight 0.68 L respirometers submerged into one of two approximately 200 L experimental tanks. Oxygen saturation (% air) inside the respirometers was continuously measured using an O_2_ optode connected to a Firesting O_2_ system (PyroScience, Aachen, Germany). A recirculation pump kept the water within the respirometer mixed, while another pump coupled to a PowerLab system (ADInstruments, Castle Hill, Australia) flushed the respirometer following automated cycles. In total, the cycles lasted 12 minutes with a 3-minute flush period, preventing the air saturation in the respirometers to go below 85%. The slope of the decline in % air saturation when the flush pump was off was used to determine MO_2_ using the following formula: MO_2_ = [(Vr—Vf) × (Δ%Sat/t) × α]/Mb; where Vr is the volume of the respirometer, Vf is the volume of the fish assuming that 1 g of tissue equals 1 ml of water, Δ%Sat/t is the change in % O_2_ saturation per time, α is the solubility coefficient of O_2_ adjusted for the respective salinities and experimental temperatures, and Mb is the body mass of the fish (Clark *et al.*, 2013).

After inserting the fish into the respirometers and commencing the cycles, MO_2_ was measured for ~ 21 hours. After the measurements the fish were removed from the respirometer and placed in a circular ~80 L tank where the fish were exhaustively exercised by manual chasing for 3 minutes and immediately transferred back to their respective respirometers, upon which the cycles resumed. This is a common practice used in order to measure maximum MO_2_ (MO_2_ max). MO_2_ was recorded for another 1–2 hours before ending the trial. At the end of each trial, several respirometry cycles were performed with empty respirometers to estimate background respiration and the average slope was subtracted from the MO_2_ slopes of the fish. The respirometers were thoroughly cleaned between trials. Standard metabolic rate (SMR) was calculated as the lower 0.20 quantile using all measurements acquired during the 23-hour trial (Chabot *et al.*, 2016). Maximum MO_2_ was the highest MO_2_ measured at any point during the whole trial (Andersson *et al.*, 2020) and aerobic scope (AS) was calculated as the difference between MO_2_ max and SMR. Note that all fish were starved for approximately 24 hours prior to being placed in respirometers.

### Liver oxidative stress analysis

#### Sample preparation

The fish were euthanized by destruction of the brain using a scalpel immediately after respirometry. Length (cm) and weight (g) were recorded for each tagged fish. Livers were excised, weighed, divided into sub-fractions, frozen in liquid nitrogen and stored at −80°C. Each liver sample was weighed and homogenized in four volumes of ice-cold buffer saline solution (0.1 M Na/K-PO4) containing 0.15 M KCl at pH 7.4 by sonication for 3 seconds. Homogenates were centrifuged for 20 minutes at 10000 rpm and 4°C. Aliquots of the supernatant (S9 fractions) were stored at −80°C until analysis.

#### Biochemical assays

All biochemical assays were measured spectrophotometrically as a change in absorbance over time (96-well plate reader Spectra Max 190 Molecular Devices) at 20°C. The enzyme activity was normalized to protein content. Total protein content was measured according to Lowry *et al.* (1951), using bovine serum albumin as standard.

*Glutathione-S-transferase (GST)* activity was measured using CDNB as substrate. 190 μl of reagent solution (120 μl CDNB 100 mM dissolved in DMSO, with 5880 μl GSH 1 mM dissolved in 0.1 M NaPO4 buffer at pH 7.5) were added to wells containing 10 μl of cytosol (diluted 10 times with homogenizing buffer) or 10 μl buffer for reference. CDNB reduction was measured at 340 nm for 3 minutes. GST activity was calculated with the extinction coefficient for glutathione-DNB adduct ε = 9600 M^−1^ cm^−1^.

*Glutathione reductase (GR)* activity was measured using oxidized glutathione (GSSG) as substrate. 160 μl of reagent solution (0.6 ml from 2.5 mg/ml NADPH and 9 ml from 4 mg/100 ml DTNB, both dissolved in 0.1 M sodium phosphate buffer pH 7.5 containing 1 mM EDTA) were added to wells containing 20 μl of cytosol (diluted 10 times with homogenizing buffer) or 20 μl buffer for reference. 20 μl of GSSG (dissolved in 0.1 M sodium phosphate buffer pH 7.5 containing 1 mM EDTA) were added to start the reaction. DTNB reduction was measured at 340 nm for 3 minutes, and activity was calculated using the extinction coefficient of TNB (ϵ = 14 151/M/cm).

*Catalase (CAT)* activity was measured using hydrogen peroxide as substrate. The degradation of hydrogen peroxide was measured at 240 nm in UV-light-sensitive plates. 190 μl of reagent solution containing 1 ml of potassium phosphate buffer solution 0.08 M pH 6.5 and 10 ml of hydrogen peroxide solution (115 μl H_2_O_2_ + 6650 μl potassium phosphate buffer) were added to wells containing 10 μl of cytosol sample in triplicates or 10 μl buffer for reference. The activity was calculated using the extinction coefficient for H_2_O_2_ (ε = 40 M^−1^ cm^−1^).

*Reduced glutathione (GSH) and oxidized glutathione (GSSG)* were measured with an indirect biochemical assay using GR. Liver samples were weighed and homogenized with exactly four times 5% SSA, sonicated for 3 seconds, precipitated on ice for 15 minutes and centrifuged for 20 minutes at 10000 rpm and 4°C. 100 μl of supernatant were saved for GSSG measurement, and 10 μl of supernatant were diluted 80× in 1.3% SSA for GSH measurement.

For GSH, 200 μl of reagent solution containing 2.5 ml 10 mM DTNB, 4.25 ml 2 mM NADPH and 18.25 ml stock buffer (143 mM NaH2PO4 + 6.3 mM EDTA, pH 7.4) was added to wells containing 20 μl of cytosol in duplicates or 20 μl standard. 20 μl of 17 IU/ml GR started the reaction. All available GSSG was converted to GSH. The absorbance of free TNB was measured at 415 nm for 7 minutes and compared to a standard curve of GSH with 10, 5, 1 and 0.5 μM diluted in acid. For GSSG, the reagent solution contained 1 mM DTNB and GSH precipitated by stirring the sample for 1 hour in RT with 5 μl of 2-vinyl pyridine. GSSG and GSH slopes were compared to standard curve slopes and calculated as μg per mg of liver weight. The ratio was calculated as GSSG/GSH × 100.

*Lipid peroxidation (LPO)* was measured with a malondialdehyde (MDA) assay kit (MAK085, Sigma-Aldrich) according to the manufacturer’s protocol. The reaction of MDA with thiobarbituric acid formed a colorimetric product that was measured at 532 nm for 1 minute. The concentration of MDA in the samples was determined from the standard curve and dilution factor and expressed in nmol/ml.

### Statistics

Statistical analyses were performed using STATISTICA 64 version 13 and R version 4.2.2 (R Core Team, 2022). Data were checked for normal distribution and homogeneity of variances prior to analysis using the Shapiro–Wilk and Levene’s test. When necessary, the data were transformed using square root, log(x) or log_10_(x + 1), and if the data remained heteroscedastic even after transformations, the non-parametic Kruskal–Wallis test was performed. One-way ANOVA or Kruskal–Wallis tests were used to test for significant differences among treatments for initial weight (g), initial length (cm), respirometry (MO_2_) SMR, respirometry maximum metabolic rate (MO_2MAX_)_,_ respirometry AS and oxidative stress enzymatic activity (GSSG, % GSSG/GSH, GSH, GR, GST, CAT and LPO). Repeated measures ANOVAs with Fisher’s (LSD) post-hoc tests were used for analysing the weight (g) and length (cm) changes over the course of the experiment (some pit-tags were lost during the exposure period and therefore not all fish could be individually identified at the end of the experiment and in such cases were not included). When ANOVA tests showed significant differences among treatments, Dunnett posteriori comparison analyses were conducted to test if treatments differed from the control. All values are means ± standard deviation (SD).

## Results

### Biological indices

The weight and length of the fish were taken at the end of the exposure period. For those with a pit-tag remaining, growth was calculated with the average end weight of 15.5 ± 5.3 g (mean ± SD) and length of 10.8 ± 1.0 cm (mean ± SD). Repeated measures ANOVA results indicated that there were no overall differences in weight at the end of the experiment among treatments (p = 0.11); however, there was a significant overall decrease in weight from the start to the end of exposure (p < 0.0001). Additionally, Fisher’s post-hoc tests (LSD) showed the low salinity (p = 0.004), high temperature (p = 0.003) and multi-stressor treatments (p = 0.038) had a significant decline in weights from the start to the end of the experiment, while that was not the case for the control or low pH treatments. There was a general slight increase in length over the course of the experiment, though not significant (p = 0.071), and no significant differences between treatments (p = 0.166). Although there was a general, non-significant increase in length over the course of the experiment, the fish in the multi-stressor treatment were the only ones that did not increase in length at all. The condition of the fish (Fulton’s condition factor K) showed no significant differences overall between treatments (p = 0.14). There was, however, a significant decrease in condition for all fish over the course of the experiment (p < 0.001).

**Figure 1:**
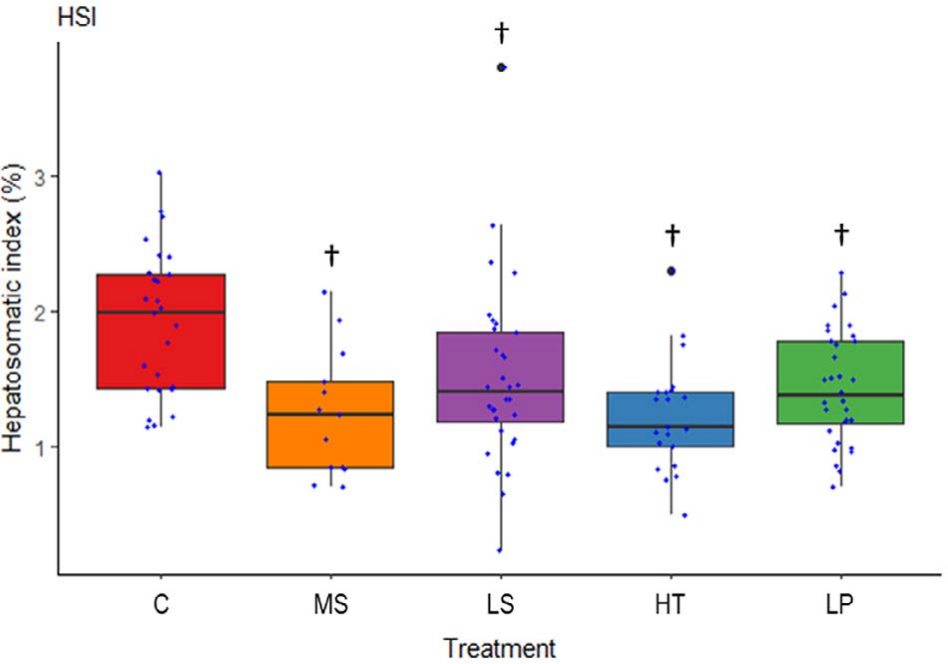
The hepatosomatic index (HSI) for the goldsinny wrasse (*Ctenolabrus rupestris*) after exposure to control conditions (C), decreased (Low) salinity (LS), increased (High) temperature (HT), decreased (Low) pH (LP) or a combination of all stressors (Multi-stressor) (MS). A significant difference (p < 0.05) from control is indicated by a † symbol. Analysis of HSI was based on square-root transformed data.

Liver weights were recorded and one-way ANOVA analysis showed significant differences between treatments (F [4, 120] = 5.43, p < 0.001) with Dunnett post-hoc analysis indicating significantly lower weight for all treatments compared to the control. From this the hepatosomatic index was calculated (HSI = 100 × (weight liver (g))/(body weight (g))) and analysis showed that HSI was significantly different among treatments (F [4, 120] = 6.57, p < 0.001) (Fig. 1). Further, the Dunnett post-hoc analysis showed that all treatments, including low salinity, high temperature, low pH and the multi-stressor treatment, had a significantly lower HSI compared to the control group (p = 0.01, p < 0.0001, p = 0.004, and p < 0.001, respectively).

### Respirometry

**Figure 2:**
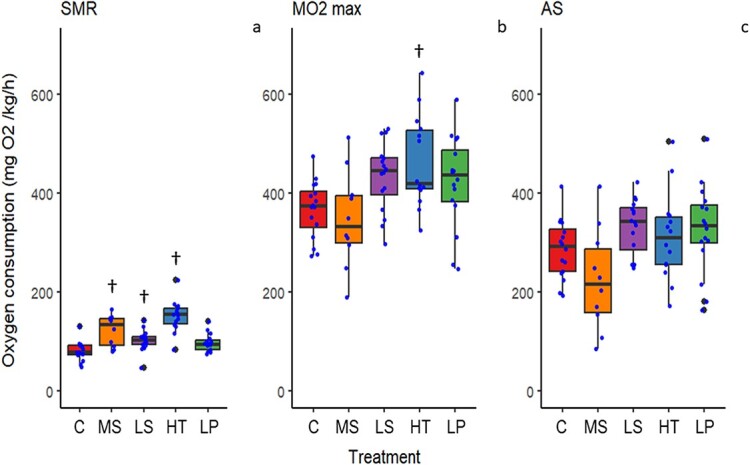
Oxygen consumption rates for the goldsinny wrasse (*Ctenolabrus rupestris*) after exposure to control conditions (C), decreased (Low) salinity (LS), increased (High) temperature (HT), decreased (Low) pH (LP) or a combination of all stressors (Multi-stressor) (MS). Left (a) is the resting, standard metabolic rate (SMR), center (b) is the maximum metabolic rate and right (c) is the calculated aerobic scope (AS). A significant difference (p < 0.05) from control is indicated by a † symbol. Analysis of SMR was based on square-root transformed data, maximum metabolic rate and aerobic scope data were untransformed for analysis.

The SMR varied significantly between treatments (p < 0.0001). Dunnett post-hoc analysis showed that fish in the low salinity, high temperature and multi-stressor treatments, all had a significantly higher SMR compared to the control treatment (p = 0.036, p < 0.0001 and p = 0.0002, respectively). The control treatment had the lowest SMR at 80.6 mg O_2_/kg/h (± 19.1), followed by the low pH treatment with a SMR of 96.0 mg O_2_/kg/h (± 18.2), the low salinity SMR of 101.3 mg O_2_/kg/h (± 21.2), and the multi-stressor SMR of 122.3 mg O_2_/kg/h (± 31.6), while the highest level was seen in the high temperature treatment with an SMR of 150.9 mg O_2_/kg/h (± 32.2) (Fig. 2 left panel).

The maximum oxygen consumption rate (MO_2_ max) also differed significantly between treatments (p = 0.003) with the Dunnett post-hoc test showing a significantly higher MO_2_ max in the high temperature treatment as compared to the control group (p = 0.008). The lowest mean was 347.0 mg O_2_/kg/h (± 97.2) in the multi-stressor treatment, followed by the control (367.2 ± 58.5 mg O_2_/kg/h), the low pH (423.3 ± 94.0 mg O_2_/kg/h), the low salinity (433.7 ± 69.4 mg O_2_/kg/h) and lastly, with the highest mean MO_2_ max exhibited by the high temperature group (463.1 ± 92.9 mg O_2_/kg/h) (Fig. 2 middle panel).

The AS showed significant differences between treatments (p = 0.011). However, the Dunnett post-hoc test did not indicate a significant difference between any of the treatments for AS in comparison to the control. The treatment with the lowest AS was the multi-stressor group (204.3 ± 119.7 mg O_2_/kg/h). The high temperature had the next lowest AS (273.2 ± 135.2 mg O_2_/kg/h), followed by the control (286.5 ± 60.1 mg O_2_/kg/h), the low pH (327.3 ± 89.4 mg O_2_/kg/h) and lastly the low salinity treatment with the highest mean AS (332.4 ± 55.8 mg O_2_/kg/h) (Fig. 2 right panel).

### Oxidative stress

**Figure 3:**
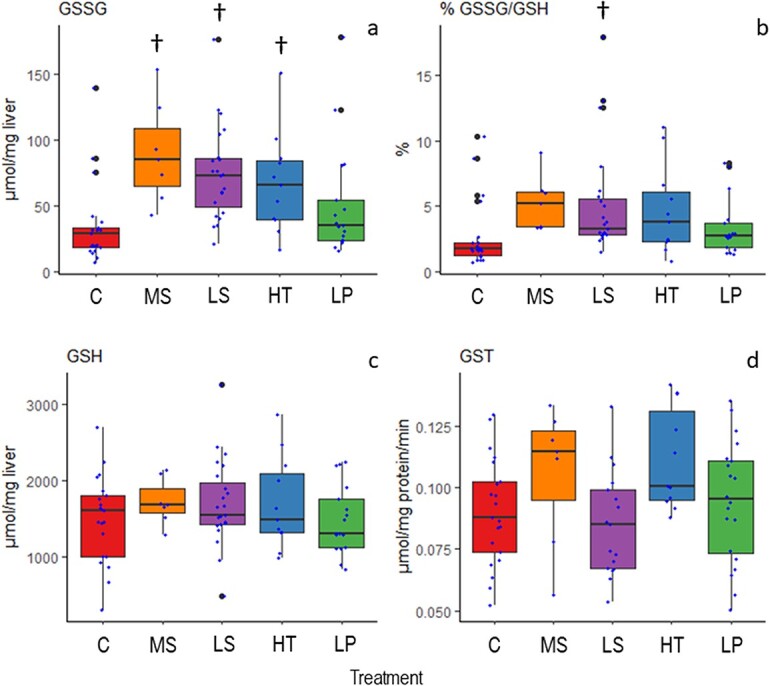
Concentration of oxidized glutathione (GSSG), reduced glutathione (GSH), ratio of oxidized glutathione (% GSSG/GSH), and glutathione-S-transferase (GST) activity in liver homogenates of goldsinny wrasse (*Ctenolabrus rupestris*) after approximately four weeks of exposure to control conditions (C), decreased (Low) salinity (LS), increased (High) temperature (HT), decreased (Low) pH (LP) or a combination of all stressors (Multi-stressor) (MS). The image on the top left (a) is GSSG, the image on the bottom left (c) is GSH, the image on the top right (b) is % GSSG/GSH and the image on the bottom right (d) is GST. The ANOVA analyses for GSSG and % GSSG/GSH were based on log-transformed data and for GSH and GST untransformed data. A significant difference (p < 0.05) from control is indicated by a † symbol.

The oxidative stress analyses of the livers showed significant differences among treatments in oxidized glutathione (GSSG) (p < 0.0001). The control had the lowest mean activity level (34.96 ± 30.80), while the multi-stressor treatment was highest (90.02 ± 38.33) (Fig. 3 top left panel). The Dunnett post-hoc analysis indicated that the low salinity, high temperature and multi-stressor treatments all differed significantly from the control group (p < 0.0001, p = 0.005, and p = 0.0002, respectively). Reduced glutathione (GSH) activity, however, was not significantly different among treatments (p = 0.528) (Fig. 3 bottom left panel). The ratio of oxidized glutathione to reduced glutathione (% GSSG/GSH) was significant (p = 0.002) (Fig. 3 top right panel). Here, the lowest values were in the control group (2.70 ± 2.62) and the highest in the multi-stressor treatment (5.23 ± 2.12). The post-hoc test indicated that for %GSSG/GSH, the low salinity and multi-stressor groups both differed significantly from the control (p = 0.001 and p = 0.008, respectively). Additionally, glutathione-S-transferase (GST) activity was also significantly different among treatments (p = 0.030). The Dunnett post-hoc test did not reveal any significant difference in the treatments from the control for GST activity, although the high temperature group was nearly so (p = 0.052), with control having the lowest and high temperature the highest mean GST level (0.0902 ± 0.022 and 0.1115 ± 0.021, respectively) (Fig. 3 bottom right panel).

**Figure 4:**
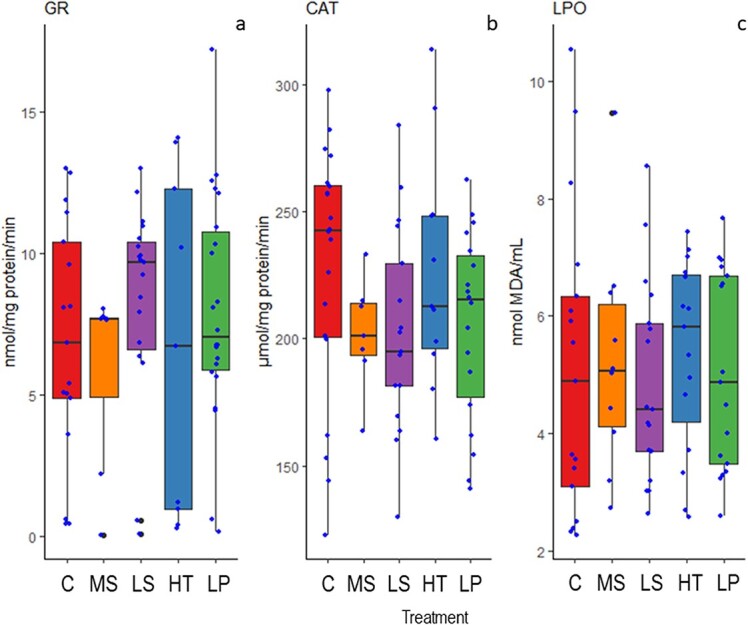
Enzymatic activities of Glutathione-reductase (GR) (a), Catalase (CAT) (b), and levels of Lipid peroxidation (LPO) (c) in the liver of goldsinny wrasse (*Ctenolabrus rupestris*) after approximately four weeks of exposure to control conditions (C), decreased (Low) salinity (LS), increased (High) temperature (HT), decreased (Low) pH (LP) or a combination of all stressors (Multi-stressor) (MS). LPO analysis was performed on square-root transformed data. A significant difference (p < 0.05) from control is indicated by a † symbol.

Additionally, glutathione-reductase (GR), catalase (CAT) and lipid peroxidation (LPO) activity in liver homogenates were analysed with no significant differences found between treatments (p = 0.584, p = 0.242 and p = 0.956, respectively) (Fig. 4).

### Fish mortality

**Figure 5:**
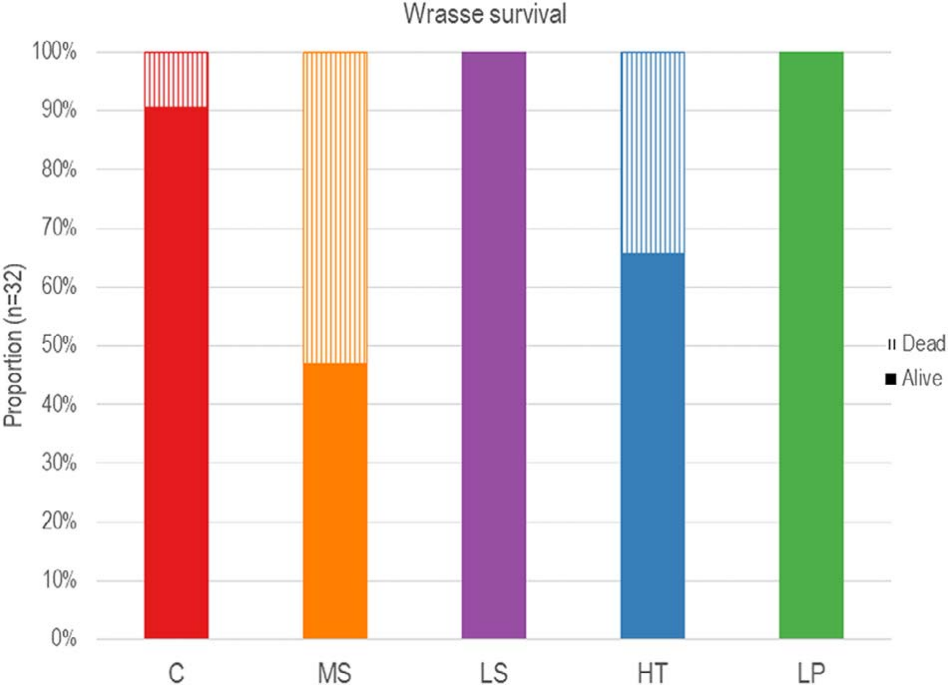
The proportion of alive (solid) and dead (striped) goldsinny wrasse (*Ctenolabrus rupestris*) individuals per treatment after exposure to control conditions (C), decreased (Low) salinity (LS), increased (High) temperature (HT), decreased (Low) pH (LP) or a combination of all stressors (Multi-stressor)(MS).

The number of deaths that occurred between treatments throughout the experiment differed significantly (H [4, 20] = 13.55, p < 0.01). Of the 160 fish included in the experiment, 31 individuals died throughout the exposure period and were therefore not included in the final measurements for growth, respirometry or oxidative stress. The control group had three deaths during exposure, so 29 individuals remained at the end for measurements. The low salinity and low pH groups had no deaths (n = 32 remaining), the high temperature had a 34% mortality rate with 11 fish dead (n = 21 remaining) and the multi-stressor group had a total of 17 individuals dead during the exposure period. Fifteen individuals remained for the final sampling, which equates to a 53% mortality rate (Fig. 5).

## Discussion

Goldsinny wrasse lives in coastal, shallow-water habitats characterized by relatively high variability of the chemical and physical properties of the surrounding water (Björk and Nordberg, 2003). It would be realistic to assume that this mesopredatory species would be adapted to wide ranges of temperature, salinity and pH (Bergström *et al*., 2016) and that the species has sufficient phenotypic plasticity to tolerate considerable environmental heterogeneity (Boyd *et al*., 2016). Nevertheless, one of the most striking results of the study presented here was the high mortality observed in the multi-stressor treatment, with over 50% of the fish within that group dying before the end of the exposure period, compared to less than 10% mortality in the control group. This finding alone is a clear indication of the deleterious effects of the cumulative impacts of future climate change on the goldsinny wrasse. In addition, we show that the hepatosomatic index (HSI), used as a proxy for energy content in fish (Dutil *et al*., 1995), was significantly lower for all treatments when compared to the control group, indicating an energetic cost related to exposure to the climate change stressors. While there were no observed effects for metabolic rate and oxidative stress of exposure to OA, there were clear indications of the physiological impacts of exposure to OF and a MHW, as well as the multi-stressor treatment. Given that the fish most likely experiencing the highest stress were found dead as a result of the cumulative impacts of the multi-stressor environment, their physiological stress parameters were not measurable. Therefore, the physiological impacts observed for respirometry and oxidative stress analyses may actually be underestimated in the multi-stressor environment due to selection of stress resistant fish.

### Physiological response

Stress responses in fish have been broken down into primary, secondary and tertiary physiological responses (Barton, 2002), where secondary stress results in metabolic and cellular changes, while tertiary stress leads to whole-animal changes such as decreases in growth and survivability (Portz *et al*., 2006). In this study, there was an observed decrease in weight over the course of the experiment in the OF, MHW and multi-stressor treatments. Additionally, liver weights and HSI were significantly lower for all treatments compared to the control meaning that exposure to OF, MHW, OA as well as the multi-stressor environment all lead to a reduction in the energy reserves stored in the liver. All of these measures are an indication of the considerable energetic demand related to coping with a stressful environment. Increased temperatures, for instance, typically cause an increase in metabolism, however, as thermal stress occurs normal physiological functions are disturbed which results in energy expended towards stress responses instead, potentially leading to death (Portz *et al*., 2006).

Overall, the single factor that had the largest effect on MO_2_ was temperature. Temperature affects the rate of all biochemical reactions (Gillooly *et al*., 2001), and wrasses in the high temperature treatment had higher SMR and MO_2_ max compared to the other treatments. We measured several different proxies for oxidative stress to detect the possible physiological changes during the experiment. The higher metabolic rates were accompanied by significantly higher concentrations of GSSG at the high temperature as well as reduced salinity and the multi-stressor environment. Increasing cellular concentrations of GSSG—a product of the oxidation of glutathione (GSH)—indicate increasing oxidative stress for the goldsinny wrasse specimens. On the other hand, there was no significant difference in the GSSG/GSH ratio when exposed to the MHW only. Normally, 98% of glutathione exists in the reduced form and any deviation from this would be considered a sign of oxidative stress (Owen and Butterfield, 2010). Moreover, none of the other measured oxidative stress dependent parameters showed elevated activities in goldsinny wrasse exposed to low salinity, high temperature or low pH. However, the fish were only exposed to the experimental conditions for 4 weeks, and it is possible that the mild oxidative stress they experience will lead to negative consequences over a longer period such as slow oxidation of macromolecules leading to accelerated senescence, as it has been shown that some fish species have a lower antioxidant capacity when they are older (reviewed by Birnie-Gauvin *et al*., 2017).

The individuals in the OF treatment had a higher SMR than those in the control. Although intuitively osmoregulatory costs should be lower at salinities closer to isosmotic conditions (Sundell *et al*., 2021), this hypothesis is often unsupported by experimental data and the relationships between SMR and salinity are highly variable and species-dependent (Ern *et al*., 2014). Species that live in coastal environments with variable conditions, such as the goldsinny wrasse, are typically osmoregulators that are able to maintain internal fluid concentrations acceptable to the animal regardless of decreasing or increasing salinity; however, as Smyth and Elliott (2016) discuss, long-term exposure to unfavourable salinities can still result in physiological consequences. Given the increased SMR, GSSG and GSSG/GSH ratio observed in the OF treatment in the current study, this appears to be the case and as Smyth and Elliott (2016) also mention this can eventually lead to death for the organism. Therefore, it can be speculated that if the current study had been prolonged the physiological stress observed due to reduced salinity may have eventually lead to mortality, although this requires further investigation to be certain. Additionally, although the OA treatment showed no significant secondary stress results for either oxygen consumption or oxidative stress, there is an indication that there is some degree of stress experienced from exposure to OA, as we do see a reduction in HSI meaning that energy reserves are beginning to be depleted and further exposure may have led to additional physiological effects.

Interestingly, because of the increases in both SMR and MO_2_ max, there did not seem to be an effect of exposure on the AS of the fish compared to the control. AS is defined as the difference between MO_2_ max and SMR and is a determinant of whole-animal performance (Pörtner and Farrell, 2008; Gräns *et al*., 2014). Temperature is frequently reported to increase MO_2_ max up to a certain temperature beyond which MO_2_ max decreases. Due to the general increase of energy demand for all physiological processes, SMR increases continuously with temperature until an optimum temperature is reached and then sharply decreases (Gianguzza *et al*., 2014). As a result of the changes to both SMR and MO_2_ max, this optimal temperature is lower than the temperature at which MO_2_ max is highest (Healy and Schulte, 2012; Gräns *et al*., 2014). Here, we observed no significant changes in AS in the high temperature treatment compared to the control treatment. There are three possible explanations for this. First, the high temperature was inside the range at which the wrasse functions optimally (though this is unlikely given the mortality observed in the MHW treatment). Optimal AS can exist within a wide range of temperatures, and it has shown considerable resilience to increasing temperature in ballan wrasse (*Labrus bergylt*), a sympatric to goldsinny wrasse (Yuen *et al*., 2019). Secondly, the fish could adequately acclimatize to the MHW temperature within the experimental period, thus pushing the optimal performance towards higher temperatures. While some degree of thermal compensation occurs with longer exposure to high temperatures, fish acclimated to higher temperatures generally maintain a higher SMR (Gräns *et al*., 2014; Sandblom *et al*., 2016). As the third possibility, subjecting the fish to the higher temperature selected for the more resilient individuals, thus increasing the average performance. The high mortality in the MHW and multi-stressor treatments does indicate that hard selection for resilience could have taken place and results from a similar experiment has shown within-treatment differences in stress response from exposure to climate change stressors (Perry *et al*., 2024). If this is the case, it would be an example of phenotypic plasticity and cryptic genotypic variation whereof different genotypes are maintained in a population with approximately equal fitness within the normal range of environments typically experienced by the species but that under extreme conditions one genotype may be better adapted to the new conditions (Ghalambor *et al*., 2007; Reed *et al*., 2010).

In efforts to understand the effects of multiple stressors on organisms and ecosystems, there has been much discussion about additive, synergistic and antagonistic effects. Meaning that the stress responses can be the sum of the response of all drivers, an effect larger than that expected from the addition of the response to single drivers by which an organism responds disproportionately strongly to the combination of drivers, or antagonistic where drivers have opposite effects that help to counteract or mitigate one another. There is also evidence that in cases of multiple drivers (stressors), there is a single dominant driver causing a severely detrimental effect and the addition of other drivers is unlikely to have an equally large effect. Notably, while many of the physiological parameters evaluated in this study show single stressor effects as well as multi-stressor effects, there is no clear indication of a single dominant stressor across all evaluated parameters together. Additionally, there are no obvious overall additive, synergistic or antagonistic effects, though there are clear cumulative effects indicated by the highest response level shown in the multi-stressor treatment for GSSG and %GSSG/GSH. There are also interesting interactive effects with the multi-stressor treatment showing a reduced MO_2_ max and AS compared to the control treatment, while each single stressor showed higher oxygen consumption rates compared to the control. With the available data, pinpointing the precise mechanisms underlying the reduced MO_2_ max in the multi-stressor treatment remains speculative as they may comprise changes affecting the oxygen transport cascade at multiple levels, including cardiac function, branchial gas exchange, tissue oxygen extraction, mitochondrial function, etc. Still, it could be suggested that the increased oxidative stress in the multi-stressor treatment, as indicated by the higher % GSSG/GSH, negatively affected one or several of these physiological processes, partly explaining the reduced MO_2_ max. Accordingly, a growing body of scientific literature suggests that combined environmental stressors (e.g. OA and MHW) often have larger deleterious effects than single stressors, and that these effects involve multiple physiological processes (Baag and Mandal, 2022). Again, although the physiological parameters show very little response to OA there are indications of stress related to OF, MHWs and the multi-stressor environment making identification of a single dominant driver difficult, the high rate of mortality observed for the MHW and multi-stressor treatments indicates a cumulative response to all stressors as well as being a clear display of the impact of increased temperature on the survival of the goldsinny wrasse.

### Ecological consequences

Given the goldsinny wrasse’s role as an important mesopredator in the marine shallow-water seascape, and the wide geographic distribution of the species (Froese and Pauly, 2023), the cumulative impacts shown in the current study have potential ecosystem-wide consequences. Although the species inhabits coastal areas which regularly experience shifts in water pH, temperature and salinity (e.g. Pihl and Rosenberg, 1982; Staveley *et al*., 2019), our results showed a reduction in HSI for OA and physiological stress responses to future OF and MHW as single stressors, and worryingly even more so when stressors are combined. Because the fish used in the current study were wild-caught, their previous exposure to environmental stressors is unknown. However, monitoring data from the region indicates that coastal habitats close to where the fish were caught have experienced an increase in environmental variability such as the documented MHW in 2018 (Anna Irma Wilcke *et al*., 2020), and therefore the adult fish used in the current study are likely to have experienced some degree of one or more of the stressors evaluated. In addition, according to a study on goldsinny wrasse in the region the species is considered mature around 2 years of age and can live between 8 and 13 years, with the average age of goldsinny caught from eight different locations being approximately 4 years (Olsen *et al*., 2018) which could mean that the adult fish in the current study may have lived through the 2018 MHW and would then be considered less sensitive to temperature changes compared to other parts of the population since they have been able to survive to adult stages. This is highly speculative, however if this was the case then the stress observed in the current study is even more worrisome on a population level. As these environmental changes are expected to continue or worsen in the future, and particularly so in boreal nearshore environments (Meier *et al*., 2022; Wåhlström *et al*., 2022), this may result in decreased resilience of the goldsinny wrasse, or even population wide declines if mortality occurs as in our study. This could potentially lead to shifts in food-web dynamics and changes in trophic cascades because this fish species is one of the most abundant species in boreal coastal habitats (Stål *et al*., 2007; Staveley *et al*., 2017; Perry *et al*., 2018b, 2018a). The goldsinny wrasse’s diet consists predominantly of amphipods and gastropods (Wennhage and Pihl, 2002; Stål *et al*., 2007) and diminished wrasse predation on these mesograzers can cause increases in filamentous algae with detrimental effects on the seagrass these algae cover causing shifts in seagrass coverage (Moksnes *et al*., 2008; Eklöf *et al*., 2012).

As well as the top-down shifts that would result from a reduction of goldsinny wrasse abundances in coastal systems, there are also potential bottom-up changes that could occur given the importance of the species for larger predators. Goldsinny wrasse is an important food source for predatory fish such as Atlantic cod and other gadoids and salmonids, as well as for coastal birds like cormorants (Härkönen, 1988; Wennhage and Pihl, 2002; Alexandersson and Lunneryd, 2005; Nedreaas *et al*., 2008). The potential cascade of effects resulting from a reduction in abundance of the goldsinny wrasse as a result of the sensitivity of the species to climate change is a cause for concern, especially given the wrasse fishery occurring in northern Europe (Andersson *et al*., 2021; Halvorsen *et al*., 2021). Removal of an important species from an already disturbed environment should be managed with extreme caution, as there are very clear examples from the region of the loss of species resilience and ecological consequences from overfishing (Cardinale and Svedäng, 2004; Casini *et al*., 2008; Baden *et al*., 2012; Bergström *et al*., 2015; Eklöf *et al*., 2020).

## Data Availability

The data underlying this article will be shared on reasonable request to the corresponding author.
